# Correlations between SARS-CoV-2 Infections and the Number of COVID-19 Vaccine Doses Administered in Three Italian Provinces

**DOI:** 10.3390/healthcare11030358

**Published:** 2023-01-27

**Authors:** Alberto Modenese

**Affiliations:** Department of Biomedical, Metabolic and Neural Sciences, University of Modena and Reggio Emilia, Via G. Campi 287, 41125 Modena, Italy; alberto.modenese@unimore.it; Tel.: +39-059-205-5475

**Keywords:** COVID-19, SARS-CoV-2, vaccinations, vaccines, ecological study

## Abstract

The aim of this ecological study is to evaluate correlations between the number of COVID-19 vaccine doses administered in three Italian provinces—one in the south, one in the center and one in the north of the country—and the registered numbers of COVID-19 cases in the same areas. The period of January 2021–September 2022 was considered, with specific analysis for fractions of times corresponding to the spread in Italy of the different SARS-CoV-2 variants. The results confirm the reduction of the effectiveness of the vaccines in preventing new COVID-19 cases in Italy, regardless of latitude, after the appearance of the first omicron variants. The new variants omicron 4 and 5 showed an extremely high spread during the Italian summer months; fortunately, the effects of the vaccinations in preventing new cases was improved compared to the previous omicron variants, showing a negative correlation between the new COVID-19 cases and the number of vaccine doses administered.

## 1. Introduction

The vaccination campaign against COVID-19 begun in Italy at the end of December 2020 [1]. There is currently a high level of scientific evidence demonstrating the significant effectiveness of the COVID-19 vaccine in preventing SARS-CoV-2 infections, considering in particular the wild-type virus spread in 2020 and the alpha and delta variants dominant in 2021 [2,3,4]. At the end of November/beginning of December 2021, the omicron variant appeared in Italy, and rapidly became the most prevalent variant in Italy during 2022 [5]. Since July 2022, the omicron 4 and 5 subtype variants have been responsible for the vast majority of Italian COVID-19 cases [6]. The effectiveness of the vaccines in preventing new and breakthrough infections has significantly decreased with the appearance of the omicron variants; fortunately, vaccines still show an important effect in preventing severe consequences of the SARS-CoV-2 infections, such as hospitalizations and deaths [7]. According to the most recent data of the Italian National Institute of Health (Istituto Superiore di Sanità, ISS), the “case fatality rate” (i.e., the number of deaths in the population of diagnosed cases) in Italy decreased from 19.6% at the beginning of the pandemic, before the beginning of the vaccination campaign, to 2.4% when the alpha variant was prevalent in January 2021, at the very beginning of vaccine administration; the rate then decreased again to 0.2% in January 2022, after the delta variant spread in Italy, reaching a minimum of 0.1% in October 2022 with the wave of omicron variant infections [8]. The number of SARS-CoV-2-infected patients requiring hospitalization has also decreased from more than 38,000 (of which about 3800 were admitted to Intensive Care Units—ICUs) in November 2020, before vaccinations, to about 32,000 (of which about 3700 were admitted to ICUs) in April 2021, with the alpha variant and a minority of the Italian population vaccinated [9]; the number of hospitalized patients decreased again to about 1300 (of which 150 were admitted to ICUs) at the time of the delta variant in July 2021, with the majority of the population having completed the primary vaccine cycle. During 2022, with a significant spread of omicron infections and a long time after the completion of the first vaccination cycle for a significant part of the population, a high number of hospitalizations occurred again, with a peak in January 2022 of about 21,500 patients hospitalized (of which 1600 were admitted to ICUs) [9]. After this peak, with a significant part of the Italian population having taken the booster dose and the “fourth dose” (i.e., the second booster, currently recommended for all people aged over 60 years old with the new bivalent COVID-19 vaccines after at least 120 days from their first booster [10]), the lowest numbers of hospitalizations and ICU admission yet have been observed in September 2022 of about 3500 and 130 cases, respectively [9].

The aim of the present study is to evaluate correlations between the number of COVID-19 vaccine doses administered in three Italian provinces and the registered numbers of COVID-19 cases. Specific sub-analyses for the periods of the pandemic corresponding to the dominancy of specific SARS-CoV-2 variants have been also conducted.

## 2. Materials and Methods

The present paper is an ecological study examining crude correlations between the number of COVID-19 vaccine doses and the reported COVID-19 cases in three Italian provinces.

The provinces of Palermo (38°06′40″ N 13°21′06″ E; 1,268,217 inhabitants), Rome (41°53′36″ N 12°28′58″ E; 4,353,738 inhabitants) and Milan (45°28′01″ N 09°11′24″ E; 3,218,201 inhabitants) were selected as, respectively, representatives for the south, the center and the north of the country.

The data of new COVID-19 cases have been registered on a daily basis in Italy since the beginning of the pandemic and reported by the Italian National Institute of Health (Istituto Superiore di Sanità, ISS [11]) and the Ministry of Health [12] for all the Italian provinces. These data were retrieved by a specific website archiving all the historical data series [13]. Moreover, the daily numbers of COVID-19 vaccine doses administered are also registered in Italy and available on the same website for each of the provinces up to the 27th November 2021 [13]. For the period 28th November 2021–September 2022, the data with details on the provinces were not available on the website [13], and we therefore collected publicly available national Italian data to be further elaborated [9]. In order to retrieve the specific data of the provinces considered, the proportion of the doses administered per province was obtained based on the total number of Italian inhabitants (60,483,973) and on the inhabitants of each province [13]. This is certainly an approximation; however, while at the beginning of the pandemic, different numbers of vaccine doses could be expected to be administered in different territories, based on the availability of the vaccines and of personnel, it is unlikely that relevant differences in administration rates could be found in these latest stages of the vaccination campaigns. All the daily doses administered were considered, not differentiating per type of vaccine nor per first vs. second or booster doses.

We tested Pearson’s r correlations in Microsoft Excel in order to correlate the data of the vaccines’ doses with the number of COVID-19 cases. We started from the 28^th^ December 2020, i.e., the first day with data on the vaccine doses administered in Italy [1,13], and we analyzed the number of vaccinations with respect to the number of new cases registered 30 days after (i.e., 27 January 2021), and this was carried out for all the subsequent days (last day considered for vaccines = 22 August 2022, correlated with the number of the COVID-19 cases registered on the 21 September 2022).

A period of 30 days was arbitrarily established to appreciate the significant effect of vaccine administration on the prevention of the registered SARS-CoV-2 infections, in order to consider a relevant immune response induced in the vaccinated subjects [14].

## 3. Results

Table 1 presents the average daily data per month related to the new cases of COVID-19 registered and the number of vaccine doses administered in the Italian provinces of Palermo, Rome and Milan from the end of December 2020 to September 2022. In the Supplementary Materials the whole dataset is reported; as indicated in the Methods section, the correspondence of dates is due to the two types of data collected (e.g., vaccine doses on the 28 December 2020 correspond to new COVID-19 cases on the 27 January 2021) (Table 1).

Considering the three provinces together, with a total population of 8,840,156, the peak of the new daily cases of SARS-CoV-2 infections was reached in March 2021 for the alpha variant, with 2606.5 new daily infections on average, corresponding to an incidence per 10,000 inhabitants of 2.95. In that month, the highest incidence of 3.50 per 10,000 was registered in Milan.

In terms of the delta variant, the peak appeared to occur in November 2021, with a total of 1235.8 and an average daily incidence of 1.40 new cases per 10,000 inhabitants, even if it cannot be excluded that the omicron 1 variant, which appeared in Italy at the end of November 2021, contributed to these numbers. In November 2021, the highest incidence was reported in Rome, with an average of 1.65 new daily cases per 10,000 inhabitants (Table 1).

Since December 2021 and until June 2022, the omicron 1, 2 and 3 variants were the most prevalent SARS-CoV-2 variants in Italy, and the highest number of new daily infections was reached in the month of January 2022, with 20,173.5 new daily cases on average considering the three Italian provinces together, representing an incidence of 22.8 cases per 100,000 inhabitants. The highest incidence in January 2022 was reported in Milan, with an average of 28.38 new cases per 10,000 inhabitants per day. Finally, in the period July–September 2022, the new omicron 4 and 5 variants spread in Italy, with a peak of 10,270.1 mean daily cases in July and an incidence of 11.62 new infections per day per 10,000 inhabitants. The highest incidence in July 2022 was reported in Rome, with a rate of 12.67 new daily cases per 10,000 inhabitants on average (Table 1).

Regarding the vaccine doses, the highest number of vaccinations were administered in the month of June 2021 (Table 1, where the data can be found in the row “July 2021”, referring to the month before for the vaccine doses administered), with an average of 204,580.4 daily doses in the three provinces, corresponding to a rate of 231.42 doses per 10,000 inhabitants; the maximum rate was in Rome, where a rate of 332.83 per 10,000 was reached. The lowest number of doses administered was registered in the month of June 2022 (Table 1, where the data can be found in the row “July 2022”, referring to the month before for the vaccine doses administered), with only 2400.0 mean daily doses, for a rate of 2.71 vaccinations per 10,000 inhabitants.

In Table 2, we present the crude correlations of new cases of COVID-19 with daily vaccine doses; overall, there is a significant negative correlation with the number of COVID-19 cases in all the three provinces, considering the entire period January 2021–September 2022 (r = −0.294). The strongest negative correlation, considering the whole period, was between the number of vaccine doses and the COVID-19 cases in Palermo, with a r of −0.425. All the correlations became stronger when considering the period of the spread of the alpha variants between January and May 2021, with a r value of −0.712 considering the three provinces together (Table 2).

With respect to delta variant cases, correlations were weaker, even if still negative in all the cases. Overall, a correlation with a r value of −0.230 was calculated. The lowest r value around 0, i.e., no correlation, was observed for Palermo province (Table 2).

For omicron variants 1–3, paradoxical positive correlations were observed for the number of vaccine doses with respect to the new cases of COVID-19, with an overall r value of 0.412 (Table 2).

Finally, the new omicron variants 4 and 5 showed negative correlations with the number of vaccine doses in all three provinces, with the highest value observed in Milan (r = −0.291), and an overall r value considering the three provinces together of −0.266 (Table 2).

## 4. Discussion

This Italian ecological study is a further demonstration of the overall effectiveness of the anti-COVID-19 vaccination campaign [2,3,4], as the number of vaccine doses administered in three Italian provinces—representing the north, the center and the south of the country— resulted in a negative correlation with the number of SARS-CoV-2 infections registered. Nevertheless, the prevention of new cases and breakthrough infections is particularly clear when observing the correlations during the first period of the study from January to May 2021, i.e., the spread of the alpha variant in Italy [15], which is more similar to the original SARS-CoV-2, used for the development of the vaccines.

The delta variants spread in Italy during summer 2021, with higher infectivity compared to alpha variants [16], and resulted in a weaker negative correlation with the number of vaccine doses administered compared to alpha. These data are most likely not only related to a reduction of the effectiveness of the vaccines in preventing new cases of delta variant infections, but can be explained by the fact that in the second half of 2021, the Italian national health system reached its maximum capacity for vaccine administrations. The highest number of subjects vaccinated in all the three provinces was more than 200,000 per day on average in June 2021, i.e., more than 20 people for every thousand inhabitants vaccinated in a single day; this was certainly a large number and due to the invaluable efforts of the national health system, but likely still insufficient to fully combat the spread of the rapidly infecting delta variants. Moreover, in this period, most of the subjects vaccinated were newly vaccinated persons, particularly young subjects not previously vaccinated, while the booster dose for fragile and elderly subjects, as well as for healthcare workers vaccinated with the primary cycle between January and April 2021, started only in September, so that many of the infections registered during summer 2021 were probably re-infections of people vaccinated three–six months before [9,13].

On the contrary, when looking at the period of the spread of the first omicron 1, 2 and 3 variants in Italy, i.e., December 2021–June 2022 [5], it seems unfortunately clear that there was a loss of effectiveness of the vaccines in preventing new COVID-19 cases and breakthrough infections. Nevertheless, the possible influence of various factors can also be considered, and in particular of the relatively low number of vaccine doses administered. Particularly in the second part of the spread of the first omicron variants in Italy, i.e., March–May 2022, a relevant decrease of the average daily doses was registered, with about 1/5 to 1/15 of the daily doses administered in the previous months, December 2021–February 2022. This is likely related to the fact that in March–May 2022, the vast majority of the people who wanted to be vaccinated against SARS-CoV-2 had already been vaccinated in the primary cycle, so that the doses administered in those months included the third booster doses of subjects who underwent the primary vaccination at a later stage and a few newly vaccinated subjects, as the campaign for the fourth booster dose was not fully started yet in Italy at that time. Moreover, the majority of the vaccinations were concentrated in the period November 2021–February 2022, because COVID-19 vaccination was proposed to be administered together with the anti-influenza vaccination, while at the end of the influenza season a rapid decrease of the daily doses administered was observed.

Finally, the data related to the recent omicron 4 and 5 variants that spread in Italy during summer 2022, are interesting [6]. It seems that the decrease of the spread of the virus registered during the summer months in Italy both in 2020 and in 2021 was not confirmed for the omicron 4 and 5 variants. The number of new daily cases was particularly high during summer 2022, especially in the provinces located at lower latitudes (i.e., Rome and Palermo). This high spread of the virus in the Italian summer months of 2022 was expected and predicted in the scientific literature, observing repeated outbreaks of COVID-19 occurring without a fixed frequency, and highlighting that no evidence of a strong seasonal pattern could be found for the trend of SARS-CoV-2 infections in Italy as well as in other countries [17].

Fortunately, negative correlations with the number of vaccine doses administered, even if quite weak, reappeared for new cases of omicron 4 and 5; this is possibly an effect of the administrations of the fourth booster vaccine doses after June 2022 to elderly and fragile subjects and healthcare workers in particular. Nevertheless, it should also be considered that the negative correlations between the most recent omicron variants infections and vaccine doses can be strengthened by the occurrence of previous SARS-CoV-2 infections, and in particular of omicron 1–3 infections [18,19].

This ecological study has various limitations: first of all, its design is unable to prove any association, but only aimed to suggest correlation patterns among variables [20]. There are additional limitations related to the data used: as indicated in the Methods section, we collected the number of daily vaccine doses administered, with no distinctions between type of vaccine and for the type of dose (i.e., first, second, boosters). Accordingly, the number of doses we used overestimated the number of subjects vaccinated. We also decided to arbitrarily consider a period of 30 days to evaluate the correlation between vaccines and COVID-19 cases; this period could be an overestimation of a possibly more appropriate period for having a positive immune response after a second or a booster dose of 15–20 days. On the other hand, it could be an underestimation in the case of a first dose, conferring only an initial protection but requiring a second dose, usually after 20 days for mRna vaccines, for a full protective effect. A further limitation related to the number of the vaccine doses is that we had to infer some of the data based on the overall national Italian data, as the specific data of the provinces were not available.

## 5. Conclusions

The results of this ecological study conducted in three Italian provinces confirm the reduction of the effectiveness of the COVID-19 vaccines in preventing new SARS-CoV-2 infections, regardless of latitude, after the appearance of the first omicron variants in Italy. The new variants omicron 4 and 5 showed a very high spread during the Italian summer months; fortunately, negative correlations between new COVID-19 cases and the number of vaccine doses have been detected, stressing the importance of continuing with the vaccination campaign, including the fourth booster doses, possibly with omicron-adapted vaccines.

## Figures and Tables

**Table 1:** Average daily COVID-19 cases and vaccine doses administered per month in Palermo, Rome and Milan in Italy from January 2021 to September 2022.

		Total Numbers (Rate Per 10,000 Inhabitants)							
		All Three Provinces		Palermo		Rome		Milan	
		COVID-19 Cases	Vaccine Doses *	COVID-19 Cases	Vaccine Doses *	COVID-19 Cases	Vaccine Doses *	COVID-19 Cases	Vaccine Doses *
Alpha variants	Jan 2021	1652.4 (1.87)	3174.8 (3.59)	287.8 (2.27)	531.6 (4.19)	883.0 (2.03)	2429.4 (5.58)	481.6 (1.50)	213.8 (0.66)
	Feb 2021	1590.4 (1.80)	22,446.2 (25.39)	229.7 (1.81)	5600.0 (44.16)	760.1 (1.75)	6378.0 (14.65)	600.6 (1.87)	10,468.2 (32.53)
	Mar 2021	2606.5 (2.95)	28,684.0 (32.45)	303.1 (2.39)	6200.5 (48.89)	1178.2 (2.71)	8764.4 (20.13)	1125.2 (3.50)	13,719.1 (42.63)
	Apr 2021	2048.1 (2.32)	67,506.2 (76.36)	407.0 (3.21)	14,717.1 (116.05)	975.5 (2.24)	21,507.4 (49.40)	665.6 (2.07)	31,281.7 (97.20)
	May 2021	859.7 (0.97)	110,528.8 (125.03)	137.3 (1.08)	21,405.1 (168.78)	434.0 (1.00)	29,589.6 (67.96)	288.4 (0.90)	59,534.1 (184.99)
Delta variants	Jun 2021	204.8 (0.23)	171,771.1 (194.31)	30.6 (0.24)	38,006.6 (299.69)	104.3 (0.24)	47,924.3 (110.08)	69.9 (0.22)	85,840.2 (266.73)
	Jul 2021	536.8 (0.61)	204,580.4 (231.42)	58.2 (0.46)	42,209.6 (332.83)	354.3 (0.81)	61,473.8 (141.20)	124.3 (0.39)	100,897.0 (313.52)
	Aug 2021	798.5 (0.90)	190,224.4 (215.18)	236.9 (1.87)	39,843.0 (314.17)	407.5 (0.94)	52,965.7 (121.66)	154.1 (0.48)	97,415.7 (302.70)
	Sep 2021	548.7 (0.62)	98,982.0 (111.97)	146.7 (1.16)	20,002.3 (157.72)	257.0 (0.59)	28,065.8 (64.46)	145.0 (0.45)	50,913.9 (158.21)
	Oct 2021	411.7 (0.47)	76,780.4 (86.85)	42.1 (0.33)	19,973.0 (157.49)	258.4 (0.59)	15,725.8 (36.12)	111.3 (0.35)	41,081.6 (127.65)
	Nov 2021	1235.8 (1.40)	51,150.1 (57.86)	83.2 (0.66)	12,409.3 (97.85)	718.6 (1.65)	14,566.2 (33.46)	434.0 (1.35)	24,174.6 (75.12)
Omicron 1–3 variants	Dec 2021	5894.4 (6.67)	67,869.5 (76.77)	306.8 (2.42)	14042.6 (110.73)	1961.3 (4.50)	21,507.2 (49.40)	3626.3 (11.27)	32,319.7 (100.43)
	Jan 2022	20,173.5 (22.8)	67,248.9 (76.07)	1788.3 (14.10)	9647.6 (76.07)	9251.6 (21.25)	33,119.8 (76.07)	9133.6 (28.38)	24,481.5 (76.07)
	Feb 2022	8403.9 (9.51)	84,580.0 (95.68)	1223.3 (9.65)	12,133.9 (95.68)	5000.3 (11.49)	41,655.3 (95.68)	2180.3 (6.77)	30,790.8 (95.68)
	Mar 2022	8631.3 (9.76)	31,135.2 (35.22)	1495.1 (11.79)	4466.7 (35.22)	4888.2 (11.23)	13,333.9 (35.22)	2248.0 (6.99)	11,334.6 (35.22)
	Apr 2022	8363.5 (9.46)	8357.4 (9.45)	1123.5 (8.86)	1199.0 (9.45)	4804.3 (11.03)	4116.0 (9.45)	2435.7 (7.57)	3042.4 (9.45)
	May 2022	4155.8 (4.70)	4228.3 (4.78)	564.6 (4.45)	606.6 (4.78)	2268.6 (5.21)	2082.4 (4.78)	1322.6 (4.11)	1539.3 (4.78)
	Jun 2022	6596.0 (7.46)	4782.2 (5.41)	995.7 (7.85)	686.1 (5.41)	3646.7 (8.38)	2355.2 (5.41)	1953.6 (6.07)	1740.9 (5.41)
Omicron 4–5 variants	Jul 2022	10,270.1 (11.62)	2400.0 (2.71)	1497.8 (11.81)	344.3 (2.71)	5515.9 (12.67)	1182.0 (2.71)	3256.4 (10.12)	873.7 (2.71)
	Aug 2022	2733.7 (3.09)	6367.5 (7.20)	422.9 (3.33)	913.5 (7.20)	1441.3 (3.31)	3136.0 (7.20)	869.5 (2.70)	2318.0 (7.20)
	Sep 2022	1954.8 (2,21)	3613.2 (4.09)	218.3 (1.72)	518.4 (4.09)	1014.4 (2.33)	1779.5 (4.09)	722.1 (2.24)	1315.4 (4.09)

* Vaccine doses refer to the previous month.

**Table 2:** Pearson’s r correlations of new SARS-CoV-2 infections with COVID-19 vaccine doses administered in Palermo, Rome and Milan in Italy from January 2021 to September 2022.

	Total Number of Vaccine Doses vs. COVID-19 Cases in All Three Provinces	Palermo Vaccine Doses vs. COVID-19 Cases	Rome Vaccine Doses vs. COVID-19 Cases	Milan Vaccine Doses vs. COVID-19 Cases
Overall correlations in the period January 2021–September 2022	−0.294	−0.425	−0.146	−0.252
Correlations in the period January–May 2021 (alpha variants)	−0.712	−0.600	−0.629	−0.676
Correlations in the period June–November 2021 (delta variants)	−0.230	−0.031	−0.200	−0.237
Correlations in the period December 2021–June 2022 (omicron 1–3 variants)	0.412	0.297	0.390	0.390
Correlations in the period July–September 2022 (omicron 4–5 variants)	−0.266	−0.121	−0.274	−0.291

## Data Availability

All the data are available in the present article and its Supplementary Materials.

## Supplementary Materials

The following supporting information can be downloaded at: https://www.mdpi.com/article/10.3390/healthcare11030358/s1, Table S1: full dataset.
